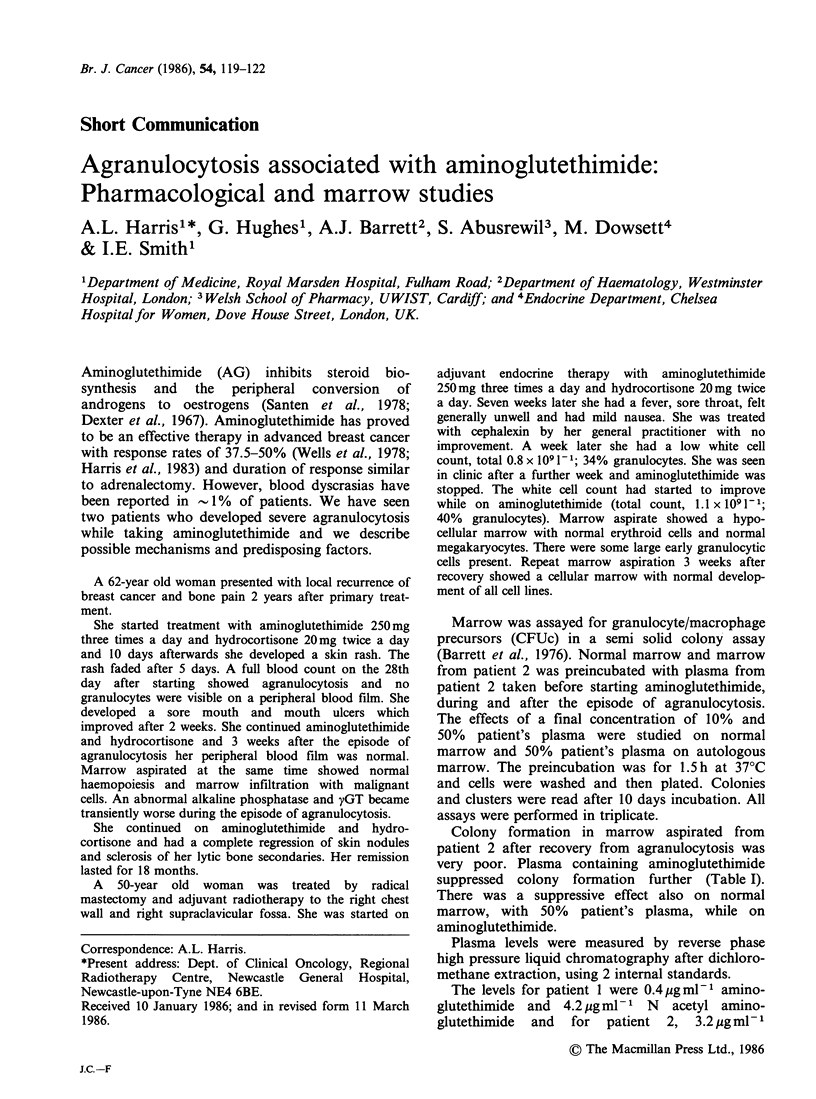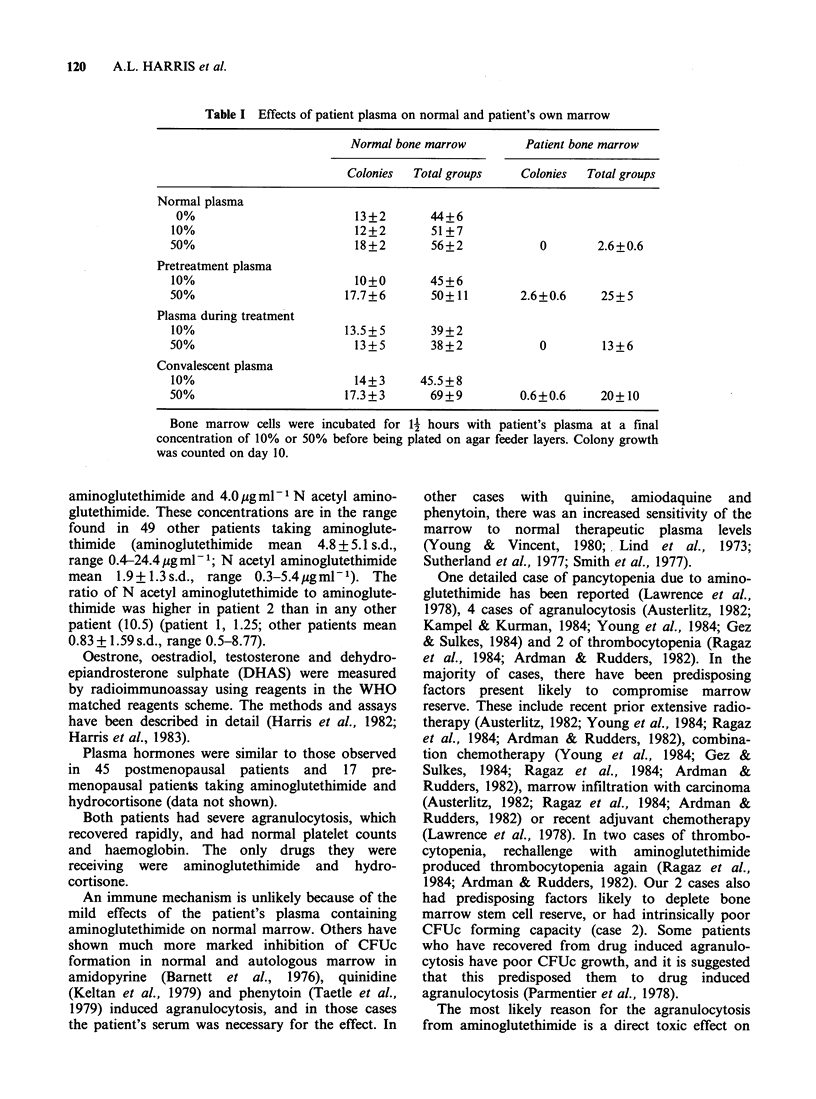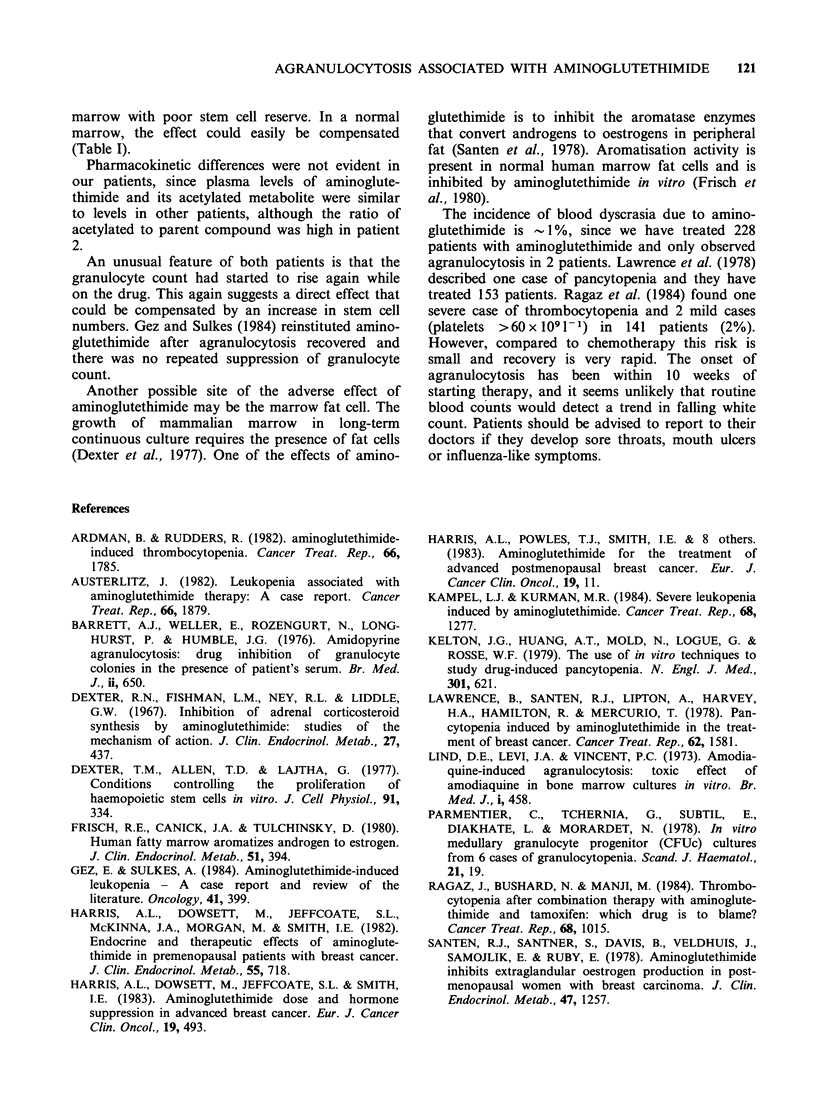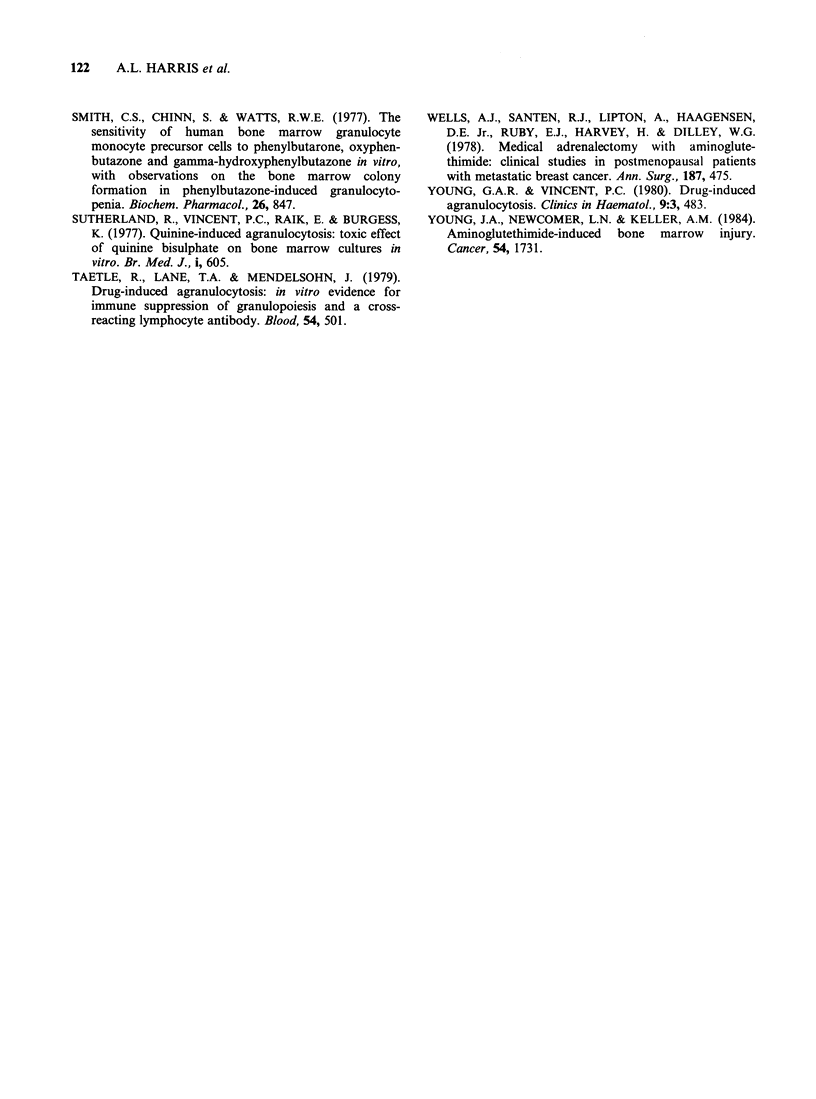# Agranulocytosis associated with aminoglutethimide: pharmacological and marrow studies.

**DOI:** 10.1038/bjc.1986.160

**Published:** 1986-07

**Authors:** A. L. Harris, G. Hughes, A. J. Barrett, S. Abusrewil, M. Dowsett, I. E. Smith


*Br. J. Cancer* (1986), **54**, 119–122

### Short Communication

### Agranulocytosis associated with aminoglutethimide: Pharmacological and marrow studies

A.L. Harris[1]*, G. Hughes[1], A.J. Barrett[2], S. Abusrewil[3], M. Dowsett[4] & I.E. Smith[1]

[1]*Department of Medicine, Royal Marsden Hospital, Fulham Road;* [2]*Department of Haematology, Westminster Hospital, London;* [3]*Welsh School of Pharmacy, UWIST, Cardiff; and* [4]*Endocrine Department, Chelsea Hospital for Women, Dove House Street, London, UK.*

Aminoglutethimide (AG) inhibits steroid biosynthesis and the peripheral conversion of androgens to oestrogens (Santen *et al.*, 1978; Dexter *et al.*, 1967). Aminoglutethimide has proved to be an effective therapy in advanced breast cancer with response rates of 37.5–50% (Wells *et al.*, 1978; Harris *et al.*, 1983) and duration of response similar to adrenalectomy. However, blood dyscrasias have been reported in ~1% of patients. We have seen two patients who developed severe agranulocytosis while taking aminoglutethimide and we describe possible mechanisms and predisposing factors.

A 62-year old woman presented with local recurrence of breast cancer and bone pain 2 years after primary treatment.

She started treatment with aminoglutethimide 250 mg three times a day and hydrocortisone 20 mg twice a day and 10 days afterwards she developed a skin rash. The rash faded after 5 days. A full blood count on the 28th day after starting showed agranulocytosis and no granulocytes were visible on a peripheral blood film. She developed a sore mouth and mouth ulcers which improved after 2 weeks. She continued aminoglutethimide and hydrocortisone and 3 weeks after the episode of agranulocytosis her peripheral blood film was normal. Marrow aspirated at the same time showed normal haemopoiesis and marrow infiltration with malignant cells. An abnormal alkaline phosphatase and $\gamma$GT became transiently worse during the episode of agranulocytosis.

She continued on aminoglutethimide and hydrocortisone and had a complete regression of skin nodules and sclerosis of her lytic bone secondaries. Her remission lasted for 18 months.

A 50-year old woman was treated by radical mastectomy and adjuvant radiotherapy to the right chest wall and right supraclavicular fossa. She was started on adjuvant endocrine therapy with aminoglutethimide 250 mg three times a day and hydrocortisone 20 mg twice a day. Seven weeks later she had a fever, sore throat, felt generally unwell and had mild nausea. She was treated with cephalexin by her general practitioner with no improvement. A week later she had a low white cell count, total $0.8 \times 10^9 l^{-1}$; 34% granulocytes. She was seen in clinic after a further week and aminoglutethimide was stopped. The white cell count had started to improve while on aminoglutethimide (total count, $1.1 \times 10^9 l^{-1}$; 40% granulocytes). Marrow aspirate showed a hypocellular marrow with normal erythroid cells and normal megakaryocytes. There were some large early granulocytic cells present. Repeat marrow aspiration 3 weeks after recovery showed a cellular marrow with normal development of all cell lines.

Marrow was assayed for granulocyte/macrophage precursors (CFUc) in a semi solid colony assay (Barrett *et al.*, 1976). Normal marrow and marrow from patient 2 was preincubated with plasma from patient 2 taken before starting aminoglutethimide, during and after the episode of agranulocytosis. The effects of a final concentration of 10% and 50% patient's plasma were studied on normal marrow and 50% patient's plasma on autologous marrow. The preincubation was for 1.5 h at 37°C and cells were washed and then plated. Colonies and clusters were read after 10 days incubation. All assays were performed in triplicate.

Colony formation in marrow aspirated from patient 2 after recovery from agranulocytosis was very poor. Plasma containing aminoglutethimide suppressed colony formation further (Table I). There was a suppressive effect also on normal marrow, with 50% patient's plasma, while on aminoglutethimide.

Plasma levels were measured by reverse phase high pressure liquid chromatography after dichloromethane extraction, using 2 internal standards.

The levels for patient 1 were $0.4 \mu g\,ml^{-1}$ aminoglutethimide and $4.2 \mu g\,ml^{-1}$ N acetyl aminoglutethimide and for patient 2, $3.2 \mu g\,ml^{-1}$

Correspondence: A.L. Harris.
*Present address: Dept. of Clinical Oncology, Regional Radiotherapy Centre, Newcastle General Hospital, Newcastle-upon-Tyne NE4 6BE.
Received 10 January 1986; and in revised form 11 March 1986.

© The Macmillan Press Ltd., 1986

**Table I** Effects of patient plasma on normal and patient's own marrow

| | *Normal bone marrow* | | *Patient bone marrow* | |
|---|---|---|---|---|
| | *Colonies* | *Total groups* | *Colonies* | *Total groups* |
| Normal plasma | | | | |
| 0% | 13±2 | 44±6 | | |
| 10% | 12±2 | 51±7 | | |
| 50% | 18±2 | 56±2 | 0 | 2.6±0.6 |
| Pretreatment plasma | | | | |
| 10% | 10±0 | 45±6 | | |
| 50% | 17.7±6 | 50±11 | 2.6±0.6 | 25±5 |
| Plasma during treatment | | | | |
| 10% | 13.5±5 | 39±2 | | |
| 50% | 13±5 | 38±2 | 0 | 13±6 |
| Convalescent plasma | | | | |
| 10% | 14±3 | 45.5±8 | | |
| 50% | 17.3±3 | 69±9 | 0.6±0.6 | 20±10 |

Bone marrow cells were incubated for 1½ hours with patient's plasma at a final concentration of 10% or 50% before being plated on agar feeder layers. Colony growth was counted on day 10.

aminoglutethimide and 4.0 $\mu$g ml$^{-1}$ N acetyl aminoglutethimide. These concentrations are in the range found in 49 other patients taking aminoglutethimide (aminoglutethimide mean 4.8±5.1 s.d., range 0.4–24.4 $\mu$g ml$^{-1}$; N acetyl aminoglutethimide mean 1.9±1.3 s.d., range 0.3–5.4 $\mu$g ml$^{-1}$). The ratio of N acetyl aminoglutethimide to aminoglutethimide was higher in patient 2 than in any other patient (10.5) (patient 1, 1.25; other patients mean 0.83±1.59 s.d., range 0.5–8.77).

Oestrone, oestradiol, testosterone and dehydroepiandrosterone sulphate (DHAS) were measured by radioimmunoassay using reagents in the WHO matched reagents scheme. The methods and assays have been described in detail (Harris *et al.*, 1982; Harris *et al.*, 1983).

Plasma hormones were similar to those observed in 45 postmenopausal patients and 17 premenopausal patients taking aminoglutethimide and hydrocortisone (data not shown).

Both patients had severe agranulocytosis, which recovered rapidly, and had normal platelet counts and haemoglobin. The only drugs they were receiving were aminoglutethimide and hydrocortisone.

An immune mechanism is unlikely because of the mild effects of the patient's plasma containing aminoglutethimide on normal marrow. Others have shown much more marked inhibition of CFUc formation in normal and autologous marrow in amidopyrine (Barnett *et al.*, 1976), quinidine (Keltan *et al.*, 1979) and phenytoin (Taetle *et al.*, 1979) induced agranulocytosis, and in those cases the patient's serum was necessary for the effect. In other cases with quinine, amiodaquine and phenytoin, there was an increased sensitivity of the marrow to normal therapeutic plasma levels (Young & Vincent, 1980; Lind *et al.*, 1973; Sutherland *et al.*, 1977; Smith *et al.*, 1977).

One detailed case of pancytopenia due to aminoglutethimide has been reported (Lawrence *et al.*, 1978), 4 cases of agranulocytosis (Austerlitz, 1982; Kampel & Kurman, 1984; Young *et al.*, 1984; Gez & Sulkes, 1984) and 2 of thrombocytopenia (Ragaz *et al.*, 1984; Ardman & Rudders, 1982). In the majority of cases, there have been predisposing factors present likely to compromise marrow reserve. These include recent prior extensive radiotherapy (Austerlitz, 1982; Young *et al.*, 1984; Ragaz *et al.*, 1984; Ardman & Rudders, 1982), combination chemotherapy (Young *et al.*, 1984; Gez & Sulkes, 1984; Ragaz *et al.*, 1984; Ardman & Rudders, 1982), marrow infiltration with carcinoma (Austerlitz, 1982; Ragaz *et al.*, 1984; Ardman & Rudders, 1982) or recent adjuvant chemotherapy (Lawrence *et al.*, 1978). In two cases of thrombocytopenia, rechallenge with aminoglutethimide produced thrombocytopenia again (Ragaz *et al.*, 1984; Ardman & Rudders, 1982). Our 2 cases also had predisposing factors likely to deplete bone marrow stem cell reserve, or had intrinsically poor CFUc forming capacity (case 2). Some patients who have recovered from drug induced agranulocytosis have poor CFUc growth, and it is suggested that this predisposed them to drug induced agranulocytosis (Parmentier *et al.*, 1978).

The most likely reason for the agranulocytosis from aminoglutethimide is a direct toxic effect on marrow with poor stem cell reserve. In a normal marrow, the effect could easily be compensated (Table I).

Pharmacokinetic differences were not evident in our patients, since plasma levels of aminoglutethimide and its acetylated metabolite were similar to levels in other patients, although the ratio of acetylated to parent compound was high in patient 2.

An unusual feature of both patients is that the granulocyte count had started to rise again while on the drug. This again suggests a direct effect that could be compensated by an increase in stem cell numbers. Gez and Sulkes (1984) reinstituted aminoglutethimide after agranulocytosis recovered and there was no repeated suppression of granulocyte count.

Another possible site of the adverse effect of aminoglutethimide may be the marrow fat cell. The growth of mammalian marrow in long-term continuous culture requires the presence of fat cells (Dexter *et al.*, 1977). One of the effects of aminoglutethimide is to inhibit the aromatase enzymes that convert androgens to oestrogens in peripheral fat (Santen *et al.*, 1978). Aromatisation activity is present in normal human marrow fat cells and is inhibited by aminoglutethimide *in vitro* (Frisch *et al.*, 1980).

The incidence of blood dyscrasia due to aminoglutethimide is $\sim 1\%$, since we have treated 228 patients with aminoglutethimide and only observed agranulocytosis in 2 patients. Lawrence *et al.* (1978) described one case of pancytopenia and they have treated 153 patients. Ragaz *et al.* (1984) found one severe case of thrombocytopenia and 2 mild cases (platelets $>60 \times 10^9 l^{-1}$) in 141 patients (2%). However, compared to chemotherapy this risk is small and recovery is very rapid. The onset of agranulocytosis has been within 10 weeks of starting therapy, and it seems unlikely that routine blood counts would detect a trend in falling white count. Patients should be advised to report to their doctors if they develop sore throats, mouth ulcers or influenza-like symptoms.

**References**

ARDMAN, B. & RUDDERS, R. (1982). aminoglutethimide-induced thrombocytopenia. *Cancer Treat. Rep.*, **66**, 1785.

AUSTERLITZ, J. (1982). Leukopenia associated with aminoglutethimide therapy: A case report. *Cancer Treat. Rep.*, **66**, 1879.

BARRETT, A.J., WELLER, E., ROZENGURT, N., LONGHURST, P. & HUMBLE, J.G. (1976). Amidopyrine agranulocytosis: drug inhibition of granulocyte colonies in the presence of patient's serum. *Br. Med. J.*, **ii**, 650.

DEXTER, R.N., FISHMAN, L.M., NEY, R.L. & LIDDLE, G.W. (1967). Inhibition of adrenal corticosteroid synthesis by aminoglutethimide: studies of the mechanism of action. *J. Clin. Endocrinol. Metab.*, **27**, 437.

DEXTER, T.M., ALLEN, T.D. & LAJTHA, G. (1977). Conditions controlling the proliferation of haemopoietic stem cells *in vitro*. *J. Cell Physiol.*, **91**, 334.

FRISCH, R.E., CANICK, J.A. & TULCHINSKY, D. (1980). Human fatty marrow aromatizes androgen to estrogen. *J. Clin. Endocrinol. Metab.*, **51**, 394.

GEZ, E. & SULKES, A. (1984). Aminoglutethimide-induced leukopenia – A case report and review of the literature. *Oncology*, **41**, 399.

HARRIS, A.L., DOWSETT, M., JEFFCOATE, S.L., McKINNA, J.A., MORGAN, M. & SMITH, I.E. (1982). Endocrine and therapeutic effects of aminoglutethimide in premenopausal patients with breast cancer. *J. Clin. Endocrinol. Metab.*, **55**, 718.

HARRIS, A.L., DOWSETT, M., JEFFCOATE, S.L. & SMITH, I.E. (1983). Aminoglutethimide dose and hormone suppression in advanced breast cancer. *Eur. J. Cancer Clin. Oncol.*, **19**, 493.

HARRIS, A.L., POWLES, T.J., SMITH, I.E. & 8 others. (1983). Aminoglutethimide for the treatment of advanced postmenopausal breast cancer. *Eur. J. Cancer Clin. Oncol.*, **19**, 11.

KAMPEL, L.J. & KURMAN, M.R. (1984). Severe leukopenia induced by aminoglutethimide. *Cancer Treat. Rep.*, **68**, 1277.

KELTON, J.G., HUANG, A.T., MOLD, N., LOGUE, G. & ROSSE, W.F. (1979). The use of *in vitro* techniques to study drug-induced pancytopenia. *N. Engl. J. Med.*, **301**, 621.

LAWRENCE, B., SANTEN, R.J., LIPTON, A., HARVEY, H.A., HAMILTON, R. & MERCURIO, T. (1978). Pancytopenia induced by aminoglutethimide in the treatment of breast cancer. *Cancer Treat. Rep.*, **62**, 1581.

LIND, D.E., LEVI, J.A. & VINCENT, P.C. (1973). Amodiaquine-induced agranulocytosis: toxic effect of amodiaquine in bone marrow cultures *in vitro*. *Br. Med. J.*, **i**, 458.

PARMENTIER, C., TCHERNIA, G., SUBTIL, E., DIAKHATE, L. & MORARDET, N. (1978). *In vitro* medullary granulocyte progenitor (CFUc) cultures from 6 cases of granulocytopenia. *Scand. J. Haematol.*, **21**, 19.

RAGAZ, J., BUSHARD, N. & MANJI, M. (1984). Thrombocytopenia after combination therapy with aminoglutethimide and tamoxifen: which drug is to blame? *Cancer Treat. Rep.*, **68**, 1015.

SANTEN, R.J., SANTNER, S., DAVIS, B., VELDHUIS, J., SAMOJLIK, E. & RUBY, E. (1978). Aminoglutethimide inhibits extraglandular oestrogen production in postmenopausal women with breast carcinoma. *J. Clin. Endocrinol. Metab.*, **47**, 1257.

SMITH, C.S., CHINN, S. & WATTS, R.W.E. (1977). The sensitivity of human bone marrow granulocyte monocyte precursor cells to phenylbutarone, oxyphenbutazone and gamma-hydroxyphenylbutazone *in vitro*, with observations on the bone marrow colony formation in phenylbutazone-induced granulocytopenia. *Biochem. Pharmacol.*, **26**, 847.

SUTHERLAND, R., VINCENT, P.C., RAIK, E. & BURGESS, K. (1977). Quinine-induced agranulocytosis: toxic effect of quinine bisulphate on bone marrow cultures *in vitro*. *Br. Med. J.*, **i**, 605.

TAETLE, R., LANE, T.A. & MENDELSOHN, J. (1979). Drug-induced agranulocytosis: *in vitro* evidence for immune suppression of granulopoiesis and a cross-reacting lymphocyte antibody. *Blood*, **54**, 501.

WELLS, A.J., SANTEN, R.J., LIPTON, A., HAAGENSEN, D.E. Jr., RUBY, E.J., HARVEY, H. & DILLEY, W.G. (1978). Medical adrenalectomy with aminoglutethimide: clinical studies in postmenopausal patients with metastatic breast cancer. *Ann. Surg.*, **187**, 475.

YOUNG, G.A.R. & VINCENT, P.C. (1980). Drug-induced agranulocytosis. *Clinics in Haematol.*, **9:3**, 483.

YOUNG, J.A., NEWCOMER, L.N. & KELLER, A.M. (1984). Aminoglutethimide-induced bone marrow injury. *Cancer*, **54**, 1731.